# Electronic Cigarettes: Are They Smoking Cessation Aids or Health Hazards?

**DOI:** 10.7759/cureus.25330

**Published:** 2022-05-25

**Authors:** Mikael Mir, Ibtisam Rauf, Sarah Goksoy, Anwar Khedr, Abbas B Jama, Hisham Mushtaq, Nitesh K Jain, Syed Anjum Khan, Salim Surani, Thoyaja Koritala

**Affiliations:** 1 Internal Medicine, University of Minnesota School of Medicine, Minneapolis, USA; 2 Internal Medicine, St. George's School of Medicine, University Centre Grenada, West Indies, GRD; 3 Internal Medicine, University of California, Berkeley, USA; 4 Critical Care Medicine, Mayo Clinic Health System, Mankato, USA; 5 Anesthesiology, Mayo Clinic, Rochester, USA; 6 Medicine, Texas A&M University, College Station, USA; 7 Medicine, University of North Texas, Dallas, USA; 8 Internal Medicine, Pulmonary Associates, Corpus Christi, USA; 9 Clinical Medicine, University of Houston, Houston, USA; 10 Internal Medicine, Mayo Clinic Health System, Mankato, USA

**Keywords:** electronic cigarettes, vuse, smoking cessation, vaping, e-cigarettes

## Abstract

The US Food and Drug Administration (FDA) recently approved the marketing of an electronic cigarette (e-cig) brand called Vuse (RJ Reynolds Vapor Company, US) to help aid in smoking cessation for adult smokers. It was believed that the consumption of traditional cigarettes and their harmful effects would be reduced given the availability of newer e-cigarettes. However, adolescent use of tobacco and nicotine products rather increased with the availability of the same e-cigarettes, and the FDA-approved market boom only worsened this problem. Although the FDA underlines the importance of marketing e-cigarettes as a possible solution for adult traditional smoking, its consequences on adolescents' health raise many concerns, which we narrated in this review article.

## Introduction and background

Electronic cigarettes (e-cigs) have been sold legally in the United States for more than a decade, but they have remained unregulated as there has not been a clear consensus surrounding the health benefits and detrimental effects of vaping. On October 12, 2021, the US Food and Drug Administration (FDA) declared for the first time the approval of the marketing of e-cigs produced by RJ Reynolds Vapor Company, US [1]. The FDA's main goal was that by authorizing e-cigs, addicted adult smokers would be compelled to use them, therefore reducing traditional cigarette consumption. Although this was a very optimistic approach to the ongoing increase of tobacco-based products, some researchers are skeptical about its effects. As young adults began consuming e-cigs with flavors, researchers were concerned that there would be severe health consequences with the approval. However, the FDA insisted that the marketing of e-cigs would be necessary for the protection of public and human health [1].

The first e-cigarette (e-cig) was patented in 2003 as a device to aid in smoking cessation and as an alternative for nicotine delivery [2]. E-cig use has grown exponentially since its inception, especially in North America [3]. Although e-cig vapor (the cloud of aerosols released by e-cigarettes) is said to be less toxic than conventional tobacco smoke, it still contains toxins due to its nicotine content, additional flavor additives, and metallic contaminants [4].

**Figure 1:**
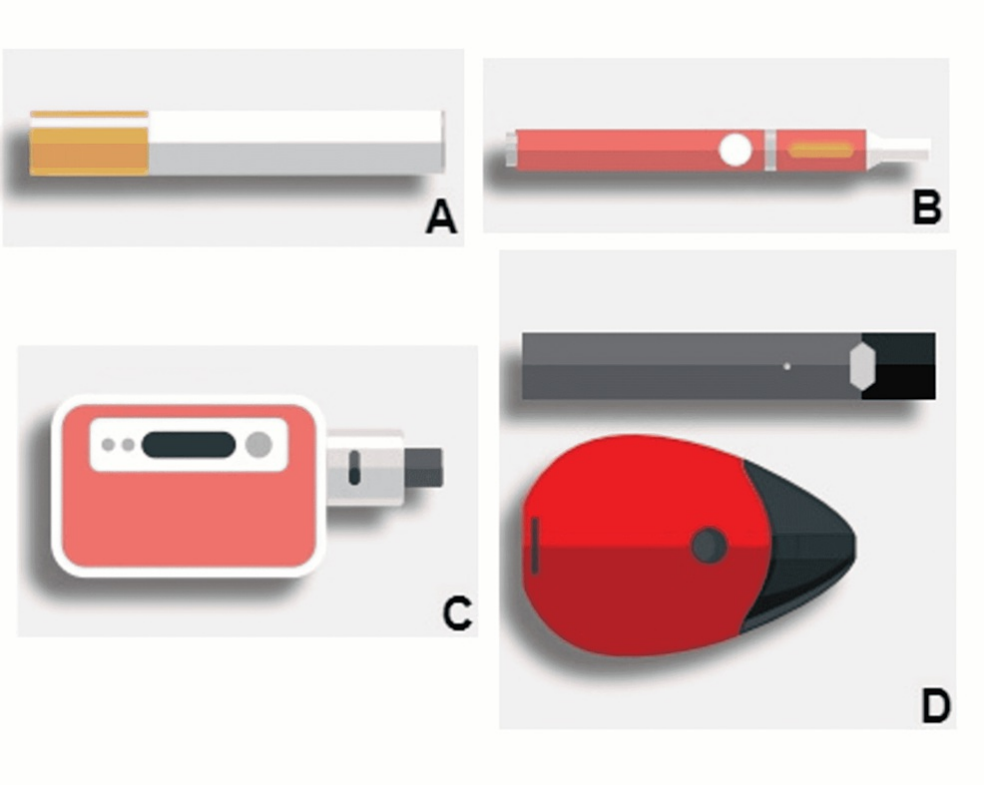
Designs of available e-cigarettes A) first generation: disposable e-cigarettes; B) second generation: e-cigarettes with prefilled or refillable cartridges; C) third generation: tanks or mods; D) fourth generation: pod mods Images developed by CDC. The images are otherwise available on the agency website for no charge. Reference to specific commercial products, manufacturers, companies, or trademarks does not constitute its endorsement or recommendation by the US Government, Department of Health and Human Services, or Centers for Disease Control and Prevention [7].

There is a standard method of e-cig delivery. Vapor is created in the e-cig through heating a solution utilized to produce the nicotine aerosol (also called e-liquid), which is inhaled by the user [5]. The e-liquid is housed in a cartridge, similar to a traditional cigarette filter, and contains an atomization chamber [6]. Depending on the brand or type of device carrier, the e-liquid contains compounds such as nicotine, propylene glycol (with or without glycerol), flavoring, and water [5,6]. The second part of the e-cig, analogous to the white paper wrapping of a traditional cigarette, contains the electronics, including the controller and battery. Users can alter many of the products of e-cigs, and there are engineering differences between brands (Table 1, Figure 1) [5,7-9], thereby altering the amount of nicotine and other chemicals delivered to the user. Though e-cigs have been on the market for decades, there is little information about their toxicity due to the lack of quality control [5]. The absorption of nicotine and other compounds in e-cigs occurs through the respiratory tract, similar to traditional cigarette smokers, and may have similar toxicokinetic properties [6]. Although, the data needed to confirm this assumption is not available at this time. 

**Table 1:** Summary of available electronic cigarettes

Generation	Brands	System type	Design
First generation: disposable e-cigarettes (Figure 1A)	VAPESTICK®, Aer Disposable, NJoy, Onejoy, Flavorvapes	Closed	Cigarette-like made to be disposable and single-use. Sometimes referred to as "ciga likes".
Second generation: e-cigarettes with prefilled or refillable cartridges (Figure 1B)	Eonsmoke, Blu, Greensmoke®, Vaporfi®, Rocket	Open	Rechargeable e-cigarettes are designed to be used multiple times. E-liquid comes in prefilled or refillable cartridges.
Third generation: tanks or mods (Figure 1C)	Halo, Volcano, Lavatube, Vaporfi®	Open	Rechargeable e-cigarettes are designed to be used multiple times. They may come with modifiable devices ("mods"), which allow the user to customize the substance in the tank.
Fourth generation: pod mods (Figure 1D)	JUUL®, Suorin, Diamond Series, V2	Open	Rechargeable e-cigarettes are designed to be used multiple times. Pod-style devices ("pods") with regulated modifications ("mods").

## Review

E-cigarette effectiveness in smoking cessation

Research regarding the efficacy of e-cigs use for smoking cessation has been done and is inconclusive. A study conducted in the United States between 2017-2019 evaluating the effectiveness of e-cigs use in smoking cessation found that subjects who substituted e-cigs for traditional cigarettes had a higher relapse rate than those who did not switch to e-cigs or any other tobacco form (adjusted risk difference 9.4%, 95% CI: 5.0%-22.8%), but this was not statistically significant. In addition, e-cigs users who switched from traditional cigarettes often tended to a high nicotine content e-cigs, negating the supposed benefit of e-cig use [10]. Furthermore, a systematic review of 38 studies found that the probability of quitting cigarettes was 28% lesser among those who utilized e-cigs compared with those who did not utilize e-cigs [11]. On the contrary, e-cig use has been shown to be more effective in smoking cessation than other nicotine replacement therapies [12], and there may be a positive correlation between e-cig use and smoking cessation. However, the low-quality data in the current published research due to the lack of well-designed randomized controlled trials and longitudinal population studies makes the evidence inconclusive [13].

While there are ample claims with regards to the effectiveness of e-cig use for smoking cessation to help individuals with addiction, e-cigs themselves can cause addiction. E-cig companies claim that their products contain less nicotine compared to traditional cigarettes, but the amount of nicotine in e-cigs is high enough to cause nicotine intoxication and addiction [14]. Although Individuals who are addicted to smoking cigarettes may find benefits in smoking cessation due to the lack of inhaled cigarette smoke, their nicotine addiction remains or even worsens [15].

The FDA's rationale for the recent marketing approval

One of the main reasons why the FDA permitted the marketing of certain e-cig products is due to the possible benefits it could have for current adult smokers with a nicotine addiction who are trying to quit using traditional cigarettes. RRJ Reynolds Vapor Company's Premarket Tobacco Product Application (PMTA) included data that indicated their tobacco-flavored products had the potential to help significantly reduce or completely eliminate the number of cigarettes consumed by current smokers [1]. The participants in the manufacturer's study were exposed to fewer harmful and potentially harmful constituents (HPHCs) from aerosols in the Vuse Solo product as compared to the participants who used traditional cigarettes. A toxicology report of the Vuse Solo's aerosols showed significantly fewer toxic substances than traditional cigarettes [1].

This new approval does not come without its stipulations. Through the PMTA pathway, e-cig manufacturers seeking similar approval must demonstrate to the FDA that their marketing strategies will be in the best interest of public health [1]. The FDA stated that they will monitor the marketing strategies of the RJ Reynolds Vapor Company to make sure they comply with the regulatory requirements of the authorization. This monitoring includes analyzing purchasing patterns and youth utilization in order to prevent developing a pattern of tobacco-flavored e-cig use [1,16]. Additionally, the RJ Reynolds Vapor Company is responsible for reporting marketing information to the FDA. Consumer research studies, advertising/marketing strategies, sales statistics, demographic data on new users, production adjustments, and negative experiences with the product are just a few examples [1]. This authorization can be taken away under the PMTA pathway if there is significant evidence indicating that other individuals who did not utilize tobacco previously, including younger populations, are using the manufacturer's product after the approval. The FDA ultimately determined the benefits to smokers that e-cigs could bring to smokers outweighed the risk of exposure to the youth if the manufacturers follow the post-marketing requirements strictly to help reduce the access and exposure of the youth to their product.

Regarding the concern of an increase in the incidence of the youth using e-cigarette products and eventually traditional cigarettes, the FDA considered the risk and consequences that this authorization could potentially bring [17]. In the 2021 National Youth Tobacco Survey (NYTS), nearly 10% of high school students who use e-cigs reported using Vuse as their go-to brand [17]. In their statement, the FDA stated that they were aware of this data and responded by highlighting the evidence indicating that younger people are less likely to use tobacco flavored electronic nicotine delivery system (ENDS) products and then switch to traditional cigarettes and are more likely to use non-tobacco flavored ENDS products. The NYTS survey data suggested that younger people are more likely to use fruit, candy, or mint-flavored, not tobacco-flavored, ENDS products, suggesting their risk of progressing to the use of a traditional cigarette is relatively low [17]. This data helped further the FDA's decision to approve Vuse marketing because the authorized tobacco-flavored products will be less appealing to the youth and more beneficial to adults who have already used traditional cigarettes. Additionally, the efforts to address youth e-cig use, including FDA's prioritized enforcement against certain unauthorized flavored e-cigs since 2020, are still ongoing [17].

The ALA's opposition to the approval

The American Lung Association (ALA) gave a public response to this decision and expressed their disappointment that the FDA is failing to meet the Tobacco Control Act's public health standard [18]. The ALA has a firm stance against the use of e-cigs for any population, warning of the irreversible lung damage and lung disease that it can cause [16]. The results of the 2021 NYTS study show that 10% of high school students who regularly utilize e-cigs use Vuse as their regular brand [17], yet the FDA continued with the approval of Vuse products. The fruity, candy, or mint-flavored ENDS products that younger populations use are part of the tobacco industry's plan for continuing the youth vaping epidemic [17]. Even if younger populations are less likely to progress to using combustible cigarettes, as mentioned in the FDA's rationale, the e-cigarettes they use still result in higher rates of nicotine addiction which poses a risk to adolescents' health [18].

The harmful effects of e-cigarette use

As a relatively new product when compared to traditional combustible cigarettes, the adverse effects of e-cig use are still being discovered. The current literature suggests multi-system consequences (Table 2) [19-44], but the respiratory system is at most risk. Since June 2019, there has been a large increase in the occurrence of e-cig/vaping-associated lung injury (EVALI) in the United States [45]. EVALI is an acute or subacute respiratory illness that can cause dyspnea, cough, hypoxemia, bilateral airspace opacities on chest imaging, and life-threatening complications that require management in the ICU [45]. EVALI is a diagnosis of exclusion and most commonly affects younger individuals, from ages 18 to 34, who, besides a significant e-cig/vaping history, are otherwise healthy [46]. In addition to EVALI, e-cigs can cause other conditions that result in hospitalization, such as vaping-induced spontaneous pneumomediastinum, and these harmful effects are being extensively studied as they present [47].

**Table 2:** Overview of e-cigarette harmful effects in both human and animal studies IL-8 - interleukin 8; H_2_O_2_ - hydrogen peroxide; COPD - chronic obstructive pulmonary disease

System	Harmful effects with mechanism
Neurological/behavioral	Amplified rewarding effects of abused drugs, cognitive decline, and emotional imbalance [19], diminished dopaminergic state [20], altered structural and neurochemical brain development in adolescents [21]
Hematological/Immunological	Cases of methemoglobinemia [22,23], leukocyte toxicity via IL-8 and H_2_O_2_ elevations leads to increased inflammatory and oxidative response [24], increased risk of head and neck cancer [25]
Respiratory	Increased susceptibility to respiratory infections [26], case of e-cigarette related organizing pneumonia [27], pro-inflammatory effects via oxidative stress [26], increased risk of developing COPD [28], diffuse alveolar damage [29], mucociliary dysfunction [30], fluctuations in surfactant composition lead to gas exchange abnormalities [31], lung damage and cell death [32], reduced functional residual capacity [33]
Cardiovascular	Increased atherosclerosis [34], increased systolic and diastolic blood pressure [35], increased heart rate [36], impaired endothelial nitric oxide synthase signaling [37]
GI/renal	Hepatocyte damage and altered nutrient metabolism [38], elevated liver biomarkers [39], disruptions and inflammation of the gut cellular barrier [40], increased susceptibility to GI infections [40], excessive collecting duct cell apoptosis [41], decreased renal function [41]
Oral	Increased pro-inflammatory markers in gingival epithelial cells [42], increased pro-senescence response in periodontal cells [42], heightened capacity for Staphylococcus aureus colonization of oral epithelial cells and biofilm formation [43], higher carriage of oral Candida albicans [44]

Even though cigarette use by younger populations has declined over the past few years, there has been an increased incidence of nicotine use from ENDS products, such as Vuse. The adolescent brain systems are known to have high plasticity [19], and the unique effects of nicotine on this plasticity are continuing to be determined. Some of the major effects are seen within the drug-reward axis, as there is a higher number and activity of nicotinic acetylcholine receptors in areas of the brain associated with reward and increased nicotine-induced dopamine release in limbic regions [19]. This drug-reward relationship leads to an increased incidence of nicotine addiction in adolescent teens, as well as behavioral changes in the rewarding effects of other abused drugs [21]. 

The harmful effects of vaping on youth aren't limited to health concerns. E-cig use affects the psychosocial, economic, and academic life of adolescents. Consuming e-cigs, especially in adolescents, impacts impulse control which can cause mood disorders and permanent damage to memory, clinical thinking, and emotional regulation [21]. As the different vaping flavors started to become popular among adolescents, a vaping culture has developed. This caused peer pressure in schools and various communities [48]. Sharing and borrowing vaping devices also contribute to an individual's social vaping identity. This social aspect of vaping in youth compels them to start or continue their use of e-cigs. In addition, there is an observed relationship between pocket money and e-cigs use. Adolescents need a higher amount of allowance to be able to buy e-cigs. This might suggest that having a higher allowance influences their smoking practice. Furthermore, vaping lowers school performance, educational attainment, and mean grades [49]. 

## Conclusions

Despite the FDA's recent approval for Vuse products to reduce the amount of traditional cigarette use in adults since e-cigs, such as Vuse, would be readily marketed and easy to access, the evidence for the effectiveness of e-cigs in smoking cessation is both preliminary and unclear. There may be some demographic factors in which e-cigs can be beneficial to smokers who are looking to quit, but there is currently minimal evidence for those cases. Additionally, the evidence surrounding the harmful effects of e-cigs use in both adolescents and adults is well documented in the literature, and more evidences are emerging regarding this area. Currently, the evidence suggests that the benefits of smoking cessation from e-cigs use do not outweigh the risks. Well-executed and randomized controlled longitudinal studies are needed before the FDA should market e-cigs as an alternative treatment option for smoking cessation, and their recent approval of the Vuse product seems premature at this time.
